# The Current State of Parkinsonism in West Africa: A Systematic Review

**DOI:** 10.1155/2021/7479423

**Published:** 2021-09-30

**Authors:** Jude T. Quarshie, Esther N. Mensah, Osbourne Quaye, Anastasia R. Aikins

**Affiliations:** ^1^Department of Biochemistry Cell and Molecular Biology, College of Basic and Applied Sciences, University of Ghana, Accra, Ghana; ^2^West African Centre for Cell Biology of Infectious Pathogens (WACCBIP), Accra, Ghana

## Abstract

Parkinsonism is one of the most common neurodegenerative diseases among the elderly. Africa is experiencing an increasing burden of age-related conditions including parkinsonism. However, there is not enough data on the prevalence, symptoms, and management of the disorder in West African patients. This systematic review examines the current state of parkinsonism in West Africa by discussing its epidemiology, symptomatology, and treatment. We searched PubMed, BioMed Central, and AJOL databases from January 2000 to December 2020 for studies on parkinsonism conducted in West African countries. We included 32 studies in this review: 23 from Nigeria, 5 from Ghana, and 1 each from Benin, Mali, Niger, and Senegal. Out of the 32 reviewed studies, 11 focused on the prevalence of parkinsonism, 4 examined the genetics of Parkinson's disease (PD), and 17 described the symptomatology and therapy of parkinsonism. The prevalence of parkinsonism in West Africa ranges from 6.0% to 8.3% of neurologic admissions/consultations. The estimated crude prevalence of PD in West Africa varies from 15 to 572 per 100,000 people. Thus far, no pathogenic genetic variants have been associated with PD in the region. Levodopa is frequently used singly or in combination with other medications to manage parkinsonian symptoms, which is consistent with reports from other African regions. Most of the reviewed studies focused only on PD, limiting assessment of other forms of parkinsonism. Almost all the prevalence studies were hospital-based and monocentric, making it impossible to accurately estimate the true prevalence of parkinsonism in West Africa. Larger community-based prevalence studies are recommended to enable accurate quantification of disease burden. Future genetic investigations should consider a wider array of gene mutations associated with parkinsonism. Moreover, public health surveillance strategies should be established to monitor the epidemiology of the disorder.

## 1. Introduction

Parkinsonism is one of the most prevalent chronic neurologic syndromes in the elderly and the second most frequent type of movement disorder after essential tremor [1]. It may be idiopathic (Parkinson's disease: PD) or may stem from underlying health conditions—collectively termed secondary parkinsonism (SP). PD, the idiopathic form of parkinsonism, is the fastest-growing neurologic disorder worldwide [2] and affects ∼2% of persons above age 60 [3–5]. There is compelling evidence that mutations at several genetic loci may mediate its development. However, disparities in findings from genetic investigations suggest that the genetics of PD is markedly different among different populations of the world [6]. For example, different variants of the *LRRK2* gene—a gene associated with PD risk—have been demonstrated to have distinct distributions in diverse populations [5]. SP may be caused by drugs, cerebrovascular accidents, manganese toxicity, and brain tumours [7–9].

Currently, Africa is experiencing rapid transitions with increased life expectancy. There is an increasing number of people aged 60 or older, and this trend is foreseen to continue [10]. Consequently, the burden of age-related conditions such as parkinsonism may be increasing [11]. Nonetheless, there is a paucity of data on the prevalence, symptoms, and management of parkinsonism disorder in African patients. Given that the disorder may result from several factors, its development may differ within African regions. This poses an important question: “what is the West African situation?” This systematic review summarizes recent studies on parkinsonism in West African countries, in order to appreciate the progress made in understanding the condition as well as emphasize the need for further research on the subject.

## 2. Methods

Online databases were systematically searched for articles on parkinsonism in West Africa published from the year 2000 to the year 2020. The databases searched were PubMed, BioMed Central, Embase, Web of Science, ScienceDirect, Scopus, and African Journals Online (AJOL). French databases “La Banque des Données en Santé Publique” (BDSP) and “Institut d'Epidémiologie Neurologique et de Neurologie Tropicale” (IENT) were also searched. Search terminologies used were [Parkinsonism OR “Parkinson's disease” OR “Parkinson disease” OR “Parkinsonian disorder”] AND “West Africa”. We also matched the term [Parkinsonism OR “Parkinson's disease” OR “Parkinson disease” OR “Parkinsonian disorder”] with all 16 West African countries to ensure all relevant studies were obtained.

Papers were initially selected based on title and abstracts, taking into account the inclusion and exclusion criteria presented below. They were then narrowed down after a critical evaluation of their full text. Publications were considered relevant if they presented data on parkinsonism in any West African country, irrespective of the study objective. References of relevant papers were manually scrutinized for publications that may have been missed in the initial search.

### 2.1. Inclusion Criteria

Original research articles, case reports, and case series on West African parkinsonian patients which were published from 2000 to 2020 formed the inclusion criteria.

### 2.2. Exclusion Criteria

Conference abstracts, editorials, short communications, systematic reviews, and meta-analyses were excluded. We also excluded publications that provided a general discussion of parkinsonism without presenting empirical data. Articles on neurologic diseases that did not present any data on parkinsonism were excluded. We excluded studies that did not undergo a formal peer-review process.

### 2.3. Data Extraction

Two reviewers (JTQ and ENM) independently conducted the data extraction from the included studies. Given the rationale of this review, we did not assess any specific outcomes. Notwithstanding, regarding the symptomatology of parkinsonism, we sought for data on primary motor symptoms, secondary motor symptoms, and nonmotor symptoms experiences by West African parkinsonian patients. We extracted information relating to the characteristics of included studies and the results are as follows:

- The report: author(s), year of publication, study setting, and study period.
- Characteristics of participants: sample size, sex, age at study, and age at disease onset.
- Study site: single- or multicentre studies and hospital- or community-based studies.
- Study design: cross-sectional, descriptive, case-control, case study, or case series.
- Eligibility criteria: criteria used to diagnose parkinsonism.
- Summary findings: prevalence and symptoms of parkinsonism, treatment regimens used.

For genetic studies, we extracted data on the genes studied, genetic mutations found, and the analytical methods used to detect mutations. Risk of bias was assessed regarding sample size, single- and multicentre studies, and referral bias.

## 3. Results

**Figure 1:**
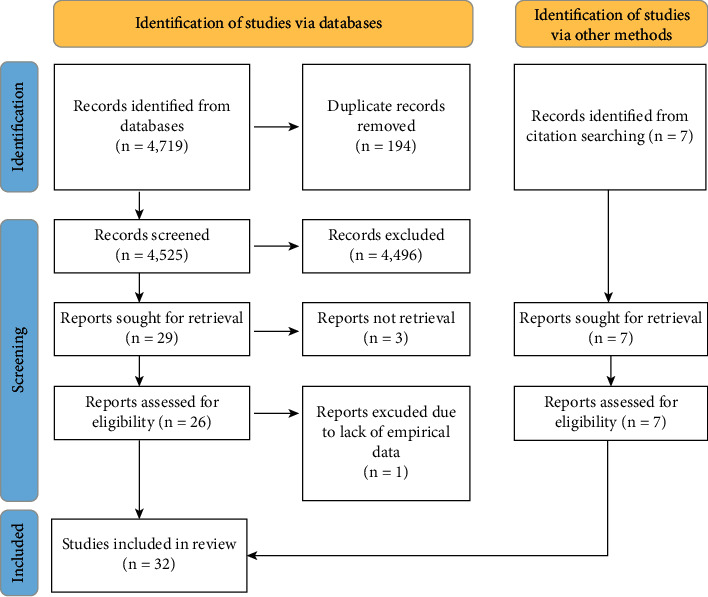
Flow diagram of literature search strategy and results.

From the nine databases searched, 29 studies were considered relevant to this review after screening of abstracts and removal of replicates. Of these, 3 articles were excluded due to unavailability of full texts, 1 paper was excluded due to lack of quantitative data, and 7 additional articles were retrieved through reference screening of selected papers, making a total of 32 articles (Figure 1).

**Figure 2:**
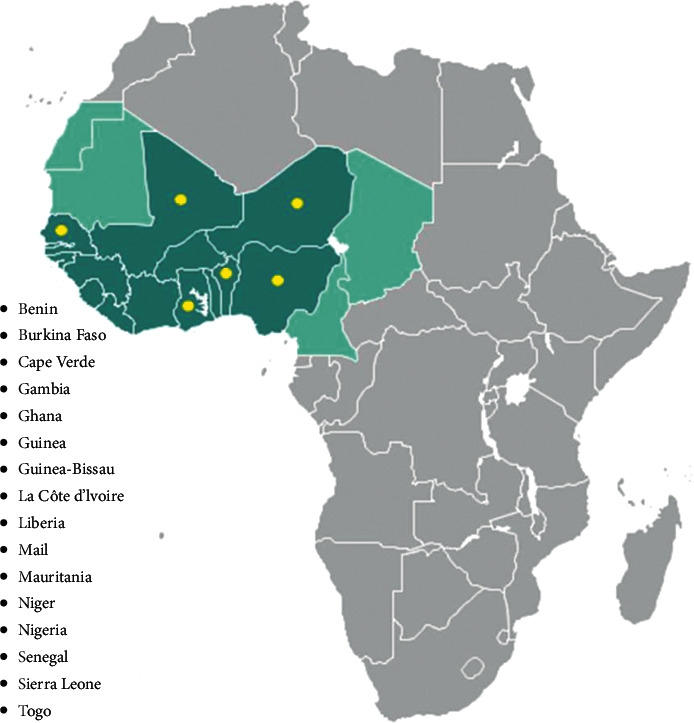
West African countries are shown in shades of green. Yellow spots represent countries from which papers were obtained. (Image from www.conceptdraw.com).

Based on the inclusion and exclusion criteria, we obtained papers from Benin, Ghana, Mali, Niger, Nigeria, and Senegal—6 of the 16 West African countries (Figure 2).

Table 1 presents an overview of the analyzed papers. Categorized by year of publication, 8 and 24 articles were published from 2000 to 2010 and from 2011 to 2020, respectively. The majority (23) of the studies were conducted in Nigeria, 5 in Ghana, and 1 each in Benin, Mali, Niger, and Senegal. All of them were conducted in a single country and almost all were monocentric studies (Table 1).

### 3.1. Prevalence of Parkinsonism in West Africa

Eleven publications described the prevalence of parkinsonism; 7 from Nigeria, 2 from Ghana, and 1 from Benin, and 1 from Niger. The majority were retrospectively conducted with all but one presenting data on the hospital prevalence of parkinsonism and/or PD. The prevalence of parkinsonism and PD varied from 6.0 to 8.3% and 0.4 to 6.9% of neurologic admissions in hospital-based studies, respectively. The only community-based survey reported a 0.09% prevalence of parkinsonism (Table 2).

For this analysis, the crude prevalence of parkinsonism or PD was calculated as the number of cases per 100,000 persons in the total population. The crude prevalence of parkinsonism, as presented in the community-based study was 91 per 100,000 people. The crude prevalence of PD varied from 15 to 572 per 100,000 people. PD was the most frequent cause of parkinsonism. Furthermore, there was a male preponderance of PD patients, with the ratio of males to females ranging from 1.5 : 1 to 4.5 : 1. The age at clinical onset of disease ranged from 22 to >67 years while the age of patients at study ranged from 50 to >68 years (Table 2).

The diagnostic criteria used for the prevalence studies were the presence of at least three of the following: tremor, rigidity, bradykinesia, and postural instability. In general, no publication directly measured predisposing factors to parkinsonism, although 2 studies reported a positive family history in some patients. The most common types of SP described were vascular parkinsonism and drug-induced parkinsonism (Table 2).

### 3.2. Genetics of PD in West Africa

Out of the 32 studies reviewed, only 4 focused on the genetics of PD, of which 3 were carried out in Nigeria and 1 in Ghana. All 4 studies examined hospital-based cohorts. One of them focused on sporadic PD cases, 2 focused on both familial and sporadic PD cases, and 1 did not specify (Table 3). A single study screened for pathogenic variants of the *ATXN3* and *PRKN* genes in Nigerian cohorts. Two groups sequenced exons 31 and 41 of the *LRRK2* gene for possible mutations, another screened for the p.G2019S mutation in the *LRRK2* gene, while the other screened for 12 mutations in the *LRRK2* gene including genetic variants classified as pathogenic in Europeans and Asians. No pathogenic variants of the studied genes were identified in any of the studies (Table 3).

### 3.3. Symptomatology and Treatment of Parkinsonism in West Africa

Seventeen publications studied the symptoms and complications of PD, the effectiveness of treatment regimens used, and the effect of dietary intake on PD treatment. Thirteen of these were conducted in Nigeria, 2 in Ghana, 1 in Mali, and 1in Senegal. All papers were hospital-based investigations, 11 were case-control studies, 3 were descriptive studies, 2 were case reports, and 1 was a comparative analysis between two groups of PD patients. There was a preponderance of male patients in the reviewed studies. The mean age at disease onset was >60 years, whereas the mean age at study was >62 years. The diagnostic criteria used were the UK Parkinson's Disease Society Brain Bank Clinical Diagnostic Criteria. None of the surveys described any predisposing factors, although, in one survey, 19 of 91 PD patients had a positive family history of the disease (Table 4).

The key motor symptoms of PD—tremor, rigidity, bradykinesia, and postural or gait abnormalities—were reported extensively in most of the reviewed literature. The secondary motor symptoms frequently experienced include freezing of gait, speech disorders, and dysphagia. The common nonmotor symptoms were cognitive and sleep disorders (Table 5). Several treatment regimens were presented in the reviewed studies. Levodopa was commonly used singly or in combination with other drugs. The dopamine agonists used were bromocriptine, pramipexole, and ropinirole whereas the anticholinergics used were trihexyphenidyl and benztropine. Only one group reported the use of an MAO inhibitor, selegiline (Table 6).

## 4. Discussion

This review represents a comprehensive summary of research data on parkinsonism in West Africa. The majority of the reviewed publications investigated only PD, and those that provided data on SP did not describe any risk factors. Nonetheless, this analysis provides data on the prevalence, symptoms, and treatment of parkinsonism in West Africa.

As expected, PD was the most diagnosed form of parkinsonism, as observed in other analyses [1]. We only present data on the estimated crude prevalence of PD in Nigeria as it was the only country with adequate data on the subject. The crude prevalence of PD varied among regions in Nigeria, as well as between different time points in the same region in the country. For example, in 2003, Talabi recorded a PD prevalence of 15 per 100,000 people in South-Western Nigeria in a 3-year retrospective study [41]. Ekenze et al., in 2010, reported a crude PD prevalence of 166 per 100,000 people in South-Eastern Nigeria in a 5-year retrospective study [40]. In 2013, Philip-Ephraim et al. recorded a crude PD prevalence of 572 per 100,000 people in South-Eastern Nigeria in a 2-year retrospective study [29]. Furthermore, Eze and Kalu in 2014 reported a crude PD prevalence of 80 per 100,000 people in South-Eastern Nigeria in a 2-year retrospective study [31]. Thus, the evidence suggests interregional variations in crude prevalence. Perhaps, estimates will less likely vary if similar methodologies and diagnostic criteria are used [43]. Nonetheless, the data we present were retrospectively collected in monocentric, hospital-based surveys, making it is difficult to accurately quantify the burden of PD within Nigeria.

The causes of SP described in the reviewed literature include drugs, cerebrovascular accidents, dementia, and head trauma. The most frequent types of SP were drug-induced and vascular parkinsonism, which is similar to the findings of other surveys [44, 45]. Drug-induced parkinsonism is the most common cause of SP in older people [1, 8] and is associated with the use of drugs that block dopaminergic receptors or deplete dopamine stores such as antipsychotics, antiemetics, and calcium channel blockers [46]. Vascular parkinsonism accounts for 4.4–12% of all parkinsonism cases and is associated with hypertension, stroke, and diabetes mellitus [45].

Although none of the studies assessed predisposing factors for PD, three articles recorded positive family history of PD in a total of 23 patients. The results of the genetic studies demonstrated the absence of mutations in the ATXN3, LRRK2, and PRKN genes amongst West Africans. Mutations in the LRRK2 gene—especially the LRRK2 G2019S mutation—are responsible for significant PD cases among North-African, European, Asian, and Middle-Eastern populations [5, 6, 47]. PD due to PRKN and ATXN3 mutations has also been described [48, 49]. The different results from genetic studies suggest that the genetics of PD is markedly different in different populations of the world and that the common LRRK2 G2019S mutation is not a frequent cause of PD in West Africa.

In all the reviewed studies, there was a preponderance of male PD patients. This observation is confirmed by previous findings which have shown that PD is more incident in males than in females, with men being at least twofold at greater risk of PD than women [50–54]. This may be attributed to the neuroprotective effect of estrogen demonstrated in females compared to males [54–56], or to the higher expression of pathogenic genes in the dopaminergic neurons of the substantia nigra pars compacta of males [50]. Other hypotheses attribute it to nonhormonal gender factors, due to the strong linkage to the X-chromosome observed in several genome-wide studies of PD susceptibility [49, 57]. Age is the most important risk factor for parkinsonism [52]. This fact is observed in this analysis where the mean age of disease onset in most studies was ≥60 years. The association between PD and age suggests that its development depends on cumulative exposure to environmental factors, and/or age-dependent biological factors [58]. Accordingly, the factors for PD etiology may be similar irrespective of geographical area or ethnicity.

The primary motor symptoms of PD—tremor, rigidity, bradykinesia, and postural or gait abnormalities—were identified in much of the reviewed literature. Some of the studies reported secondary motor symptoms such as micrographia, hypomimia, speech problems, and dysphagia. Additionally, nonmotor symptoms like cognitive disorders, sleep disorders, and gastrointestinal disorders were observed in some studies. Consistent with other studies, levodopa was used in combination with other drugs such as dopamine agonists to manage parkinsonian symptoms. Other drugs mentioned were anticholinergics and monoamine oxidase inhibitors, both of which have been shown to alleviate the symptoms of PD [52]. A recent study by Hamid et al. demonstrated that PD-specific therapies are largely unavailable and unaffordable in most African countries [59]. There is a need to initiate and drive policies aimed at improving accessibility to treatment.

### 4.1. Strengths and Limitations

This review, to the best of our knowledge, is the first of its kind on parkinsonism in West Africa. The information presented in this analysis will inform decisions regarding the development of future parkinsonism screening strategies. Although the data we provide is robust due to the systematic method of article evaluation, there are limitations to this study. Firstly, due to a lack of sufficient data, the epidemiology of parkinsonism in other countries apart from Ghana and Nigeria could not be analyzed. Again, most of the surveys were retrospective and focused only on PD. Furthermore, almost all prevalence studies were hospital-based and monocentric, making it impossible to accurately estimate the true prevalence of parkinsonism in West Africa.

## 5. Conclusion

Although research on parkinsonism in West Africa has increased over the years, there is still inadequate data on its epidemiology. PD in West Africa occurs more in males and usually begins after age 60. Moreover, a substantial number of patients experience cognitive dysfunction, motor fluctuations, sleep disorders, and reduced pulmonary function. More community-based epidemiological surveys should assess the risk factors of the disease. Moreover, public health surveillance strategies should be established to monitor the epidemiology of the disorder.

## Figures and Tables

**Table 1:** Overview of reviewed publications.

Categories	Subcategories	Number of publications	References
Year of publication	2016 to 2020	10	[12–21]
	2011 to 2015	14	[22–35]
	2006 to 2010	6	[7], [36–40]
	2000 to 2005	2	[41,42]

Country	Benin	1	[19]
	Ghana	5	[13, 15, 25, 27, 32]
	Mali	1	[21]
	Niger	1	[17]
	Nigeria	23	[7, 12, 14, 18, 20, 22–24, 26, 28–31, 33–42]
	Senegal	1	[16]

**Table 2:** Summary of prevalence studies.

Author(s), reference	Country, geographic region	Setting, study design, study period	Population, characteristics	Diagnostic criteria used	Crude prevalence of PKS and PD	Summary findings	Study limitations
Talabi [41]	Nigeria, South-Western	Hospital, cross-sectional, 1998–2000	Population: 26,355 Neurologic cases: 781 PD cases: 4 (0.5%)	NA	PD: 15	PD is a rare cause of admission	1. Single-centre study 2. Diagnostic criteria undefined 3. Prevalence data reflects hospital prevalence but not true prevalence of population. 4. Risk of referral bias as study site is a tertiary facility.

Ekenze et al. [40]	Nigeria, South-Eastern	Hospital, cross-sectional, 2003–2007	Population: 8440 Neurologic cases: 1249 PD cases: 14 (1.1%) Males: 10 (71.4%) Females: 4 (28.6%) Male-female ratio = 2.5 : 1 Peak age incidence >70 y	NA	PD: 166	NA	1. Single-centre study 2. Diagnostic criteria undefined 3. Prevalence data reflects hospital prevalence but not true prevalence of population. 4. Risk of referral bias as study site is a tertiary facility.
Okubadejo et al. [7]	Nigeria, South-Western	Hospital, cross-sectional, 1996–2006	PKS cases: 124 PD cases: 98 Males: 75 (76.5%) Females: 23 (23.5%) Male-female ratio = 3.3 : 1 Age at PD onset [mean ± SD (range)]: 61.5 ± 10.0 (37–77) y 2°PKS cases: 26 Males: 19 (73.1%) Females: 7 (26.9%) Male-female ratio = 2.7 : 1 Age at 2°PKS onset [mean ± SD (range)]: 57.5 ± 14.0 (22–78) y	Presence of at least three of the following: tremors, rigidity, bradykinesia, and postural or gait abnormality	NA	One (1) PD patient had a family history of PD in a first-degree relative. Causes of 2°PKS: ^*∗*^Vascular parkinsonism ^*∗*^Drug-induced parkinsonism ^*∗*^Multiple system atrophy ^*∗*^Lewy body dementia ^*∗*^Carbon-monoxide poisoning ^*∗*^Progressive supranuclear palsy ^*∗*^Hemiparkinsonism-hemiatrophy ^*∗*^Juvenile parkinsonism with dystonia and hemiatrophy	1. Single-centre study 2. Risk of referral bias as study site is a tertiary facility.

Owolabi et al. [39]	Nigeria, North-Western	Hospital, cross-sectional, 2005–2007	Population: 6282 Neurologic cases: 980 PD cases: 4 (0.4%) Males: 4 (100.0%) Females: 0 (0.0%) Age range at study: 50–68 y	Presence of at least three of the following: tremors, rigidity, akinesia or bradykinesia, and postural instability	PD: 63	NA	1. Single-centre study 2. Prevalence data reflects hospital prevalence but not true prevalence of population. 3. Risk of referral bias as study site is a tertiary facility.
Femi et al. [24]	Nigeria, North-Western	Hospital, cross-sectional, 2007–2011	Neurologic cases: 1153 PD cases: 80 (6.9%) Males: 61 (76.3%) Females: 19 (23.7%) Male-female ratio = 3.2 : 1 Age at PD onset [mean ± SD (range)]: 58.2 ± 6.72 (39–76) y 2°PKS cases: 16 (1.4%) Males: 13 (81.3%) Females: 3 (18.7%) Male-female ratio = 4.3 : 1 Age at 2°PKS onset [mean ± SD (range)]: 51.4 ± 10.04 (30–67) y	Presence of at least three of the following: tremors, rigidity, bradykinesia, and postural or gait abnormality	NA	Three (3) PD patients had a family history of PD in a first-degree relative. Causes of 2°PKS: ^*∗*^Vascular parkinsonism ^*∗*^Drug-induced parkinsonism ^*∗*^Head trauma-related parkinsonism	1. Risk of referral bias as study site is a tertiary facility.

Philip-Ephraim et al. [29]	Nigeria, South-Eastern	Hospital, cross-sectional, 2009–2010	Population: 699 Neurologic cases: 152 PD cases: 4 (2.6%) Males: 4 (100.0%) Females: 0 (0.0%)	NA	PD: 572	NA	1. Single-centre study 2. Diagnostic criteria undefined 3. Prevalence data reflects hospital prevalence but not true prevalence of population. 4. Risk of referral bias as study site is a tertiary facility.
Eze and Kalu [31]	Nigeria, South-Eastern	Hospital, cross-sectional, 2012-2013	Population: 1247 Neurologic cases: 267 PD cases: 1 (0.4%) Males: 1 (100.0%) Females: 0 (0.0%)	NA	PD: 80	NA	1. Single-centre study 2. Diagnostic criteria undefined 3. Prevalence data reflects hospital prevalence but not true prevalence of population. 4. Risk of referral bias as study site is a tertiary facility.

Sarfo et al. [15]	Ghana, Southern	Hospital, cross-sectional, 2011–2013	Neurologic cases: 1836 PKS cases: 120 (6.5%) Male-female ratio = 2.1 : 1 Age at study [median (IQR)]: 65 (58–74) y PD cases: 102 (5.6%)	NA	NA	Causes of 2°PKS: ^*∗*^Vascular parkinsonism ^*∗*^Parkinson plus syndromes (multiple system atrophy)	1. Single-centre study 2. Diagnostic criteria undefined 3. Risk of referral bias as study site is a tertiary facility.

Sarfo et al. [13]	Ghana, Southern	Hospital, cross-sectional, 2008–2013	Neurologic cases: 6494 PD cases: 33 (0.5%) Male-female ratio = 4.5 : 1 Age at study [mean ± SD]: 70.6 ± 11.5 y	NA	NA	NA	1. Single-centre study 2. Diagnostic criteria undefined 3. Risk of referral bias as study site is a tertiary facility.

Assadeck et al. [17]	Niger	Hospital, cross-sectional, 2009–2013	Neurologic cases: 1695 2°PKS cases: 76 (4.5%) PD cases: 25 (1.5%) Males: 15 (60%) Females: 10 (40%) Male-female ratio = 1.5 : 1 Age at onset [mean (range)]: 58 (42–74) y	NA	NA	Low (1.47%) frequency of PD	1. Single-centre study 2. Diagnostic criteria undefined 3. Risk of referral bias as study site is a tertiary facility
Adoukonou et al. [19]	Benin, Northern	Community cross-sectional Jun.–Aug. 2014	Population: 1094 PKS cases: 1 (0.09%)	Presence of akineto-hypertonic syndrome and/or resting tremor and nasal-palpebral reflex inexhaustible	PKS: 91	Cause of PKS: drug-induced	1. Risk of information bias as age was self-reported in a population with high illiteracy rate 2. Subjective signs such as pain and paresthesia may have been exaggerated or minimized.

NA: not available; crude prevalence: is given as (cases/100,000) × population; PD: Parkinson's disease; PKS: parkinsonism; 2°PKS: secondary parkinsonism; y: years; SD**:** standard deviation; IQR: interquartile range.

**Table 3:** Summary of genetic studies.

Author(s), reference	Country	Population characteristics	Genes studied	Method of analysis	Summary findings	Study limitations
Okubadejo et al. [38]	Nigeria	PD cases: 57 Males: 43 (75.4%) Females: 14 (24.6%) Age at study [mean ± SD (range)]: 62.3 ± 9.1 (43–80) y	*ATXN3* *LRRK2* *PRKN*	Screen for pathogenic repeat expansions Sanger sequencing of exons 31 and 41 Sanger sequencing of all exons and exon/intron boundaries	No pathogenic expansions No variants Several variants but none pathogenic	Small sample size; thus, definite conclusions about the prevalence of gene mutations are unachievable.

Cilia et al. [25]	Ghana	PD cases: 54 Males: 33 (61.1%) Females: 21 (75.4%) Age at study [mean ± SD (range)]: 65 ± 12 (34–89) y Age at onset [mean ± SD (range)]: 59.5 ± 12 (30–83) y	*LRRK2*	Sequencing of exon 31 and exon 41 with their intron-exon boundaries	One nonpathogenic variant	Small sample size; thus, definite conclusions about the prevalence of gene mutations are unachievable.

Okubadejo et al. [18]	Nigeria	PD cases: 123 Males: 93 (73.8%) Females: 3 (26.2%) Age at study [mean ± SD (range)]: 61.9 ± 9.9 (36–81) y Age at onset [mean ± SD]: 59.0 ± 13 y	*LRRK2*	Kompetitive Allele Specific PCR (KASP) assay to screen for p.G2019S mutation	*LRRK2* p.G2019S mutation is not implicated in PD in Nigerian patients	1. Other known mutations of the LRRK2 gene were not screened. 2. Screening method did not allow for the identification of novel pathologic variants.

Rizig et al. [12]	Nigeria	PD cases: 92 Males: 70 (76.1%) Females: 22 (23.9%) Age at study [mean ± SD (range)]: 62.1 ± 9.2 (39–90) y Age at onset [mean ± SD (range)]: 58.8 ± 9.4 (36–78) y	*LRRK2*	Kompetitive Allele Specific PCR (KASP) assay of 12 variants	*LRRK2* pathogenic alleles were absent for all 12 SNPs	1. Other known mutations of the LRRK2 gene were not screened. 2. Screening method did not allow for the identification of novel pathologic variants.

PD: Parkinson's disease; ATXN3: ataxin 3; LRRK2: leucine-rich repeat kinase 2; PRKN: Parkin RBR E3 Ubiquitin Protein Ligase; SNPs: single nucleotide polymorphisms; PCR: polymerase chain reaction; y: years; SD: standard deviation.

**Table 4:** Summary of other studies.

Author(s), reference	Country, geographic region	Setting, study design, study period	Population characteristics	Diagnostic criteria used	Summary findings	Study limitations
Okubadejo et al. [42]	Nigeria, South-Western	Hospital, case-control	PD cases: 33 Males: 25 (75.8%) Females: 8 (24.2%) Age at study [mean ± SD (range)]: 63.2 ± 10.2 (39–80) y Age at onset [mean ± SD]: 60.6 ± 10.3 y	Presence of at least three of the following: tremors, rigidity, bradykinesia, and postural or gait abnormality	Parasympathetic dysfunction occurs in PD, majority of which is symptomatic. Age >65 is associated with parasympathetic dysfunction in PD	1. Single-centre study 2. Small sample size 3. Risk of referral bias as study site is a tertiary facility.

Alasia et al. [36]	Nigeria, Southern	Hospital, case report	PD case: 1 Males: 1 (100%) Age at study: 71 y		Toxic (septic) parkinsonism may be induced by gram negative septicaemia	1. Small sample size. 2. Causative bacteria not stated.

Akinyemi et al. [37]	Nigeria, South-Western	Hospital, case-control, Jul.–Dec. 2005	PD cases: 51 Males: 37 (72.6%) Females: 14 (27.4%) Age at study [mean ± SD (range)]: 65.1 ± 9.2 (42–85) y Age at onset [mean ± SD (range)]: 60.9 ± 8.4 (41–80) y	UK Parkinson's disease society brain bank clinical diagnostic criteria.	Cognitive dysfunction is associated with old age, late PD onset, and higher disease severity Late PD onset is an independent predictor of cognitive dysfunction.	1. Single-centre study 2. Small sample size 3. Risk of referral bias as study site is a tertiary facility 4. All subjects had rest tremors, which may constitute a selection bias as akinetic-predominant PD subjects may suffer from worse cognitive impairment than tremor-predominant

Ojo et al. [22]	Nigeria, South-Western	Hospital, case-control, Mar.–Sept. 2006	PD cases: 40 Males: 32 (80%) Females: 8 (20%) Age at study [mean ± SD]: 65.8 ± 9.8 y	UK Parkinson's disease society brain bank clinical diagnostic criteria.	Hyperhomocysteinaemia is common in PD patients with prolonged use of LD but has no relationship with disease severity or disability	1. Small sample size. 2. Long-term follow-up of the different variables was not done. 3. Risk of information bias as patient's self-reported compliance and dosages used. 4. Other causes of hyperhomocysteinaemia were not evaluated

Ojo et al. [23]	Nigeria, South-Western	Hospital, case-control, Jan.–Sept. 2006	PD cases: 40 Males: 32 (80%) Females: 8 (20%) Age at study [mean ± SD]: 65.8 ± 9.8 y	UK Parkinson's disease society brain bank clinical diagnostic criteria.	Cognitive impairment and depression in PD are related to disability and worsening disease severity.	1. Small sample size. 2. Risk of referral bias as study site is a tertiary facility.
Barichella et al. [27]	Ghana. multiregional	Hospital, case-control	PD cases: 55 37 (67.3%) males 18 (32.7%) females Age at study [mean ± SD]: 65.8 ± 10.5 y	UK Parkinson's disease society brain bank clinical diagnostic criteria.	Daily intake of protein in Ghanaian patients with PD positively influences their response to LD treatment	1. Small sample size

Ojagbemi [26]	Nigeria, South-Western	Hospital, comparative	PD cases: 50 Males: 28 (56%) Females: 22 (44%) Age at study [mean ± SD]: 64.3 ± 9.7 y Age at onset [mean ± SD]: 62.1 ± 10.2 y	UK Parkinson's disease society brain bank clinical diagnostic criteria.	Hallucinations and agitations differentiate PD patients with cognitive dysfunction from those with normal cognition.	1. Small sample size 2. Single-centre study 3. Possibility of misclassification of SP as PD 4. Risk of information bias as caregivers who reported patients' symptoms may have exaggerated or understated symptoms.

Ojagbemi et al. [28]	Nigeria, South-Western	Hospital, case-control, Jul.–Dec. 2009	PD cases: 50 Males: 28 (56%) Females: 22 (44%) Age at study [mean ± SD]: 65.4 ± 9.4 y Age at onset [mean ± SD]: 62.1 ± 10.2 y	UK Parkinson's disease society brain bank clinical diagnostic criteria.	Neuropsychiatric symptoms occur frequently in PD	1. Risk of information bias as caregivers who reported patients' symptoms may have exaggerated or understated symptoms. 2. Possibility of misclassification of SP as PD 3. Risk of referral bias as study site is a tertiary facility. 4. Drug treatment may have increased risk of neuropsychiatric symptoms in PD patients than controls

Okunoye and Asekomeh [30]	Nigeria, Southern	Hospital, case-control, Jun.–Nov. 2009	PD cases: 36 Males: 27 (75%) Females: 9 (25%) Age at study [mean ± SD]: 64.3 ± 10.9 y	UK Parkinson's disease society brain bank clinical diagnostic criteria.	Depression may be common among patients with PD	1. Small sample size 2. Single-centre study
Cilia et al. [32]	Ghana, multiregional	Hospital, case-control, 2008–2012	PKS cases: 101 Primary atypical PKS cases: 2 2°PKS cases: 8 PD cases: 91 Males: 58 (63.7%) Females: 33 (36.3%) Male-female ratio = 1.8 : 1 Age at PKS onset [mean ± SD (range)]: 60.6 ± 11.3 (27–91) y	Presence of at least three of the following: tremors, rigidity, bradykinesia, and postural or gait abnormality	Nineteen (19) PD patients had a positive family history. Motor fluctuations and dyskinesias are not associated with the duration of LD therapy, but rather with longer disease duration and higher LD daily dose	1. Control group had different genetic and environmental background. 2. Access to medications between the study and control groups complicated the study design. 3. Risk of inclusion bias as the study was a hospital-based trial.

Okunoye [33]	Nigeria, Southern	Hospital, descriptive, Jun.–Nov. 2009	PD cases: 36 Males: 27 (75%) Females: 9 (25%)	UK Parkinson's disease society brain bank clinical diagnostic criteria.	Nonmotor symptoms occur in PD	1. Small sample size 2. Single-centre study

Okunoye et al. [35]	Nigeria, Southern	Hospital, case-control	PD cases: 36 Males: 27 (75%) Females: 9 (25%) Age at study [mean ± SD]: 64.3 ± 10.9 y Age at onset [mean ± SD (range)]: 60.8 ± 10.5 (39–80) y Age at onset [mean ± SD (range)]: 60.8 ± 10.5 (39–80) y.	UK Parkinson's disease society brain bank clinical diagnostic criteria.	Patients with PD have much poorer generic and specific health related quality of life in comparison to their healthy counterparts	1. Small sample size 2. Single-centre study 3. Questionnaire lacked items on self-image, night time sleep problems, sexual activity, and finances which were major concerns for patients.

Owolabi et al. [34]	Nigeria, North-Western	Hospital, case-control	PD cases: 80 Age at study [mean ± SD (range)]: 61.1 ± 8.5 y	UK Parkinson's disease society brain bank clinical diagnostic criteria.	Significant features of gastrointestinal dysfunction in PD include constipation, sialorrhea, dysphagia, difficult mastication, and choking.	1. Risk of referral bias as study site is a tertiary facility.
Maiga et al. [16]	Senegal	Hospital, descriptive, Apr.–Jun. 2014	PD cases: 35 Males: 21 (60%) Females: 4 (40%) Age at study [mean ± SD (range)]: 65.7 ± 7.4 (48–79) y Age at onset [mean ± SD (range)]: 63 ± 7.89 (46–77) y	UK Parkinson's disease society brain bank clinical diagnostic criteria.	Major alteration of sleep quality occurs in PD.	1. Scales used were available in the local language; thus, they were probably not adapted to the sociocultural context of Senegalese populations.

Owolabi et al. [14]	Nigeria, North-Western	Hospital, case-control	PD cases: 78 Males: 60 (76.9%) Females: 18 (23.1%) Age at study [mean ± SD]: 62.32 ± 8.67 y	UK Parkinson's disease society brain bank clinical diagnostic criteria.	Pulmonary function is reduced in PD	1. Pulmonary function parameters that are conventionally used to evaluate respiratory muscle strength were not employed

Ekpe [20]	Nigeria, South-Eastern	Hospital, case report	PD case: 1 Males: 1 (100%) Age at study: 72 y		Severe vomiting and diarrhoea could be symptoms of PD	1. Small sample size

Maïga et al. [21]	Mali	Hospital, descriptive, Jan.–Nov. 2013	PD cases: 60 Males: 41 (68.3%) Females: 19 (31.7%) Age at study [mean (range)]: 66.5 4 (25–94) y	UK Parkinson's disease society brain bank clinical diagnostic criteria.	Nonmotor symptoms of PD include sensitive disorders, dysautonomia, psychobehavioral disorders, and sleep disorders	1. Lack of complete patient data.

PD: Parkinson's disease; PKS: parkinsonism; 2°PKS: secondary parkinsonism; LD: levodopa; y: years; SD: standard deviation.

**Table 5:** Symptoms and signs of PD described.

Category	Symptom or sign	Brief description	References
Primary motor symptoms	Resting tremor	Slight tremor in the hand or foot on one side of the body	[7, 16, 17, 20, 24, 26, 32, 37]
	Bradykinesia	Difficulty with repetitive movements	[17, 20, 24, 26, 32, 37]
	Rigidity	Stiffness and inflexibility of the limbs, neck, and trunk	[7, 16, 17, 20, 24, 26, 37]
	Postural instability	Tendency to be unstable when standing upright	[17, 24, 37]

Secondary motor symptoms	Freezing of gait	Hesitating before stepping and exaggerated first step	[17, 24, 32]
	Micrographia	Shrinkage in handwriting	[24]
	Hypomimia	Decreased expressiveness of the face	[24]
	Falls	Falling due to instability	[24, 32]
	Speech problem	Dysarthria, drooling, and excess saliva	[17, 24, 30, 33, 37]
	Dysphagia	Difficulty in swallowing	[22, 25, 29, 30, 34]

Nonmotor symptoms	Cognitive disorders	Delusion, hallucination, depression, agitation, anxiety, apathy, anxiety, irritability, forgetfulness	[17, 21, 23, 24, 26, 28, 30, 32, 33]
	Pain	Generalised body pains	[16, 17, 21, 24, 33]
	Sleep disorder	Insomnia, interrupted sleep, excessive daytime sleepiness	[16, 17, 21, 24, 33, 37]
	Autonomic dysfunction	Cardiovascular disorders	[42]
	Pulmonary problems	Reduced vital capacity	[14]
	Sialorrhea	Excessive salivation	[21, 34]
	Mood disorder	Persistent low mood	[33, 37]
	Sweating	Excessive sweating	[17, 33]
	Genitourinary disorders	Urinary incontinence, urinary urgency	[21]
	Gastrointestinal disorders	Constipation, indigestion, and abdominal pain	[17, 20–22, 25, 34]

**Table 6:** Medications recorded in publications.

Drugs used	References
Levodopa	[16, 22, 26, 32]
Dopamine agonist	[7, 16, 24]
Anticholinergic	[17, 24]
Monoamine oxidase inhibitor	[24]
Levodopa + carbidopa	[7, 14, 15, 17, 23, 24]
Levodopa + dopamine agonist	[16, 22, 26]
Levodopa + anticholinergic	[22, 26]
Levodopa + carbidopa + anticholinergic	[7, 23]
Levodopa + dopamine agonist + anticholinergic	[7, 22, 26]

## Data Availability

The data for the study were obtained from the public databases: PubMed, BioMed Central, Embase, Web of Science, ScienceDirect, Scopus, African Journals Online (AJOL), “La Banque des Données en Santé Publique” (BDSP), and “Institut d'Epidémiologie Neurologique et de Neurologie Tropicale” (IENT).

## References

[1] Barbosa M. T., Caramelli P., Maia D. P. (2006). Parkinsonism and Parkinson’s disease in the elderly: a community-based survey in Brazil (the bambuí study). *Movement Disorders*.

[2] GBD 2016 Parkinson’s Disease Collaborators (2018). Global, regional, and national burden of Parkinson’s disease, 1990–2016: a systematic analysis for the global burden of disease study 2016. *Lancet Neurology*.

[3] Iddi S., Li D., Aisen P. S. (2018). Estimating the evolution of disease in the Parkinson’s progression markers initiative. *Neurodegenerative Diseases*.

[4] Oluwole O. G., Kuivaniemi H., Carr J. A. (2019). Parkinson’s disease in Nigeria: a review of published studies and recommendations for future research. *Parkinsonism & Related Disorders*.

[5] Shu L., Zhang Y., Sun Q., Pan H., Tang B. (2019). A comprehensive analysis of population differences in LRRK2 variant distribution in Parkinson’s disease. *Frontiers in Aging Neuroscience*.

[6] Yonova-Doing E., Atadzhanov M., Quadri M. (2012). Analysis of LRRK2, SNCA, Parkin, PINK1, and DJ-1 in zambian patients with Parkinson’s disease. *Parkinsonism & Related Disorders*.

[7] Okubadejo N. U., Ojo O. O., Oshinaike O. O. (2010). Clinical profile of parkinsonism and Parkinson’s disease in Lagos, Southwestern Nigeria. *BMC Neurology*.

[8] Skogar O., Nilsson M. (2018). Distribution of non-motor symptoms in idiopathic Parkinson’s disease and secondary parkinsonism. *Journal of Multidisciplinary Healthcare*.

[9] Al-Janabi W., Zaman I., Memon A. (2019). Secondary parkinsonism due to a large anterior cranial fossa meningioma. *European Journal of Case reports in Internal Medicine*.

[10] Velkoff V., Kowal P. (2007). Population aging in Sub-Saharan Africa: demographic dimensions 2006. *Current Population Reports, P95/07-1 Population*.

[11] Lekoubou A., Echouffo-Tcheugui J. B., Kengne A. P. (2014). Epidemiology of neurodegenerative diseases in sub-Saharan Africa: a systematic review. *BMC Public Health*.

[12] Rizig M., Ojo O., Athanasiou-Fragkouli A. (2020). Negative screening for 12 rare LRRK2 pathogenic variants in a cohort of Nigerians with Parkinson’s disease. *Neurobiology Aging*.

[13] Sarfo F. S., Awuah D. O., Nkyi C., Akassi J., Opare-Sem O. K., Ovbiagele B. (2016). Recent patterns and predictors of neurological mortality among hospitalized patients in central Ghana. *Journal of the Neurological Sciences*.

[14] Owolabi L., Nagoda M., Babashani M. (2016). Pulmonary function tests in patients with Parkinson’s disease: a case-control study. *Nigerian Journal of Clinical Practice*.

[15] Sarfo F. S., Akassi J., Badu E., Okorozo A., Ovbiagele B., Akpalu A. (2016). Profile of neurological disorders in an adult neurology clinic in Kumasi, Ghana. *eNeurologicalScience*.

[16] Maiga B., Diop M. S., Sangare M. (2016). Sleep quality assessment in 35 Parkinson’s disease patients in the fann teaching hospital, Dakar, Senegal. *Revue Neurologique*.

[17] Assadeck H., Daouda M. T., Djibo F. H., Maiga D. D., Omar E. A. (2018). Clinical profile of Parkinson’s disease: experience of Niger. *Journal of Neurosciences in Rural Practice*.

[18] Okubadejo N. U., Rizig M., Ojo O. O. (2018). Leucine rich repeat kinase 2 (LRRK2) GLY2019SER mutation is absent in a second cohort of Nigerian Africans with parkinson disease. *PLoS One*.

[19] Adoukonou T., Adogblé L., Agbétou M., Gnonlonfoun D. D., Houinato D., Ouendo E. M. (2020). Prevalence of the major neurological disorders in a semi-urban community in Northern Benin. *eNeurologicalScience*.

[20] Ekpe L. (2016). Severe vomiting and diarrhea in a parkinson disease patient: case report and review of relevant literatures. *Case Study and Case Report*.

[21] Maïga B., Koné A., Landouré G. (2016). Non-motor signs in patients with Parkinson’s disease at the university hospital of point “G,” Mali. *eNeurologicalScience*.

[22] Ojo O. O., Oladipo O. O., Ojini F. I., Sanya E. O., Danesi M. A., Okubadejo N. U. (2011). Plasma homocysteine level and its relationship to clinical profile in Parkinson’s disease patients at the Lagos university teaching hospital. *West African Journal of Medicine*.

[23] Ojo O., Okubadejo N., Danesi M., Ojini F. (2012). Frequency of cognitive impairment and depression in Parkinson′s disease: a preliminary case-control study. *Nigerian Medical Journal*.

[24] Femi O. L., Ibrahim A., Aliyu S. (2012). Clinical profile of parkinsonian disorders in the tropics: experience at Kano, Northwestern Nigeria. *Journal of Neurosciences in Rural Practice*.

[25] Cilia R., Sironi F., Akpalu A. (2012). Screening LRRK2 gene mutations in patients with Parkinson’s disease in Ghana. *Journal of Neurology*.

[26] Ojagbemi A. (2013). Relationship between cognitive dysfunction and behavioural symptoms in Nigerian patients with Parkinson’s disease no dementia. *Journal of Parkinson’s Disease*.

[27] Barichella M., Akpalu A., Cham M. (2013). Nutritional status and dietary habits in Parkinson’s disease patients in Ghana. *Nutrition*.

[28] Ojagbemi A. A., Akinyemi R. O., Baiyewu O. (2013). Neuropsychiatric symptoms in Nigerian patients with Parkinson’s disease. *Acta Neurologica Scandinavica*.

[29] Philip-Ephraim E. E., Eyong K. I., Chinenye S., William U. E., Ephraim R. P. (2013). The burden of inpatient neurologic disease in a tropical african hospital. *Canadian Journal of Neurological Sciences/Journal Canadien des Sciences Neurologiques*.

[30] Okunoye O., Asekomeh G. (2013). Depression among patients with parkinson’s disease in a Nigerian tertiary hospital. *Nigerian Health Journal*.

[31] Eze C., Kalu U. (2014). Pattern of neurological admissions in the tropics: experience at Abakaliki South-Eastern Nigeria. *Journal of Biology Agriculture and Healthcare*.

[32] Cilia R., Akpalu A., Sarfo F. S. (2014). The modern pre-levodopa era of Parkinson’s disease: insights into motor complications from sub-Saharan Africa. *Brain*.

[33] Okunoye O. (2014). Non-motor features in Parkinson’s disease patients attending neurology clinic at a tertiary Institution in Nigeria: a preliminary report. *Nigerian Health Journal*.

[34] Owolabi L. F., Samaila A. A., Sunmonu T. (2014). Gastrointestinal complications in newly diagnosed Parkinson’s disease: a case-control study. *Tropical Gastroenterology: Official Journal of the Digestive Diseases Foundation*.

[35] Okunoye O., Asekomeh G., Owolabi M., Onwuchekwal A., Ogunniyi A. (2014). Profile of generic and disease-specific health-related quality of life among Nigerians with Parkinson’s disease. *Nigerian Health Journal*.

[36] Alasia D. D., Asekomeh G. A., Unachuku C. N. (2006). Parkinsonism induced by sepsis: a case report. *Nigerian Journal of Medicine: Journal of the National Association of Resident Doctors of Nigeria*.

[37] Akinyemi R. O., Okubadejo N. N., Akinyemi J. O., Owolabi M. O., Owolabi L. F., Ogunniyi A. (2008). Cognitive dysfunction in Nigerians with Parkinson’s disease. *Movement Disorders*.

[38] Okubadejo N., Britton A., Crews C. (2008). Analysis of Nigerians with apparently sporadic parkinson disease for mutations in LRRK2, PRKN and ATXN3. *PLoS One*.

[39] Owolabi L., Shehu M., Shehu M., Fadare J. (2010). Pattern of neurological admissions in the tropics: experience at Kano, Northwestern Nigeria. *Annals of Indian Academy of Neurology*.

[40] Ekenze O. S., Onwuekwe I. O., Ezeala Adikaibe B. A. (2010). Profile of neurological admissions at the university of Nigeria teaching hospital Enugu. *Nigerian Journal of Medicine: Journal of the National Association of Resident Doctors of Nigeria*.

[41] Talabi O. A. (2003). A 3 year review of neurologic admissions in university college hospital Ibadan, Nigeria. *West African Journal of Medicine*.

[42] Okubadejo N. U., Danesi M. A. (2004). Frequency and predictors of autonomic dysfunction in Parkinson’s disease: a study of African patients in Lagos, Nigeria. *The Nigerian Postgraduate Medical Journal*.

[43] Taba P., Asser T. (2002). Prevalence of Parkinson’s disease in Estonia. *Acta Neurologica Scandinavica*.

[44] Amod F. H., Bhigjee A. I. (2019). Clinical series of Parkinson’s disease in KwaZulu-Natal, South Africa: retrospective chart review. *Journal of the Neurological Sciences*.

[45] Thanvi B., Lo N., Robinson T. (2005). Vascular parkinsonism—an important cause of parkinsonism in older people. *Age and Ageing*.

[46] Kim J. S., Youn J., Shin H., Cho J. W. (2013). Nonmotor symptoms in drug-induced parkinsonism and drug-naïve parkinson disease. *Canadian Journal of Neurological Sciences/Journal Canadien des Sciences Neurologiques*.

[47] Ishihara L., Gibson R. A., Warren L. (2007). Screening for Lrrk2 G2019S and clinical comparison of Tunisian and North American caucasian Parkinson’s disease families. *Movement Disorders*.

[48] Gwinn-Hardy K., Singleton A., O’Suilleabhain P. (2001). Spinocerebellar ataxia type 3 phenotypically resembling parkinson disease in a black family. *Archives of Neurology*.

[49] Scott W. K., Nance M. A., Watts R. L. (2001). Complete genomic screen in parkinson disease. *Journal of the American Medical Association*.

[50] Cantuti-Castelvetri I., Keller-Mcgandy C., Bouzou B. (2007). Effects of gender on nigral gene expression and parkinson disease. *Neurobiology of Disease*.

[51] Verma A., Raj J., Sharma V., Singh T., Srivastava S., Srivastava R. (2016). Epidemiology and associated risk factors of parkinson’s disease among the North Indian population. *Clinical Epidemiology and Global Health*.

[52] Balestrino R., Schapira A. H. V. (2020). Parkinson disease. *European Journal of Neurology*.

[53] Gwinn-Hardy K., Chen J. Y., Liu H. C. (2000). Spinocerebellar ataxia type 2 with parkinsonism in ethnic Chinese. *Neurology*.

[54] Balash Y., Korczyn A. D., Migirov A. A., Gurevich T. (2019). Quality of life in Parkinson’s disease: a gender‐specific perspective. *Acta Neurologica Scandinavica*.

[55] Murray H. E., Pillai A. V., Mcarthur S. R. (2003). Dose- and sex-dependent effects of the neurotoxin 6-hydroxydopamine on the nigrostriatal dopaminergic pathway of adult rats: differential actions of estrogen in males and females. *Neuroscience*.

[56] Leranth C., Roth R. H., Elsworth J. D., Naftolin F., Horvath T. L., Redmond D. E. (2000). Estrogen is essential for maintaining nigrostriatal dopamine neurons in primates: implications for Parkinson’s disease and memory. *The Journal of Neuroscience*.

[57] Pankratz N., Nichols W., Uniacke S. (2003). Genome-wide linkage analysis and evidence of gene-by-gene interactions in a sample of 362 multiplex parkinson disease families. *Human Molecular Genetics*.

[58] Wickremaratchi M. M., Ben-Shlomo Y., Morris H. R. (2009). The effect of onset age on the clinical features of Parkinson’s disease. *European Journal of Neurology*.

[59] Hamid E., Ayele B., Massi D. (2021). Availability of therapies and services for Parkinson’s disease in Africa: A Continent-Wide Survey. *Movement Disorders*.

